# Neurological and cardiopulmonary manifestations of pulmonary arteriovenous malformations

**DOI:** 10.3389/fmed.2024.1449496

**Published:** 2024-09-19

**Authors:** Weida Lu, Honggang Dai, Yunyi Li, Xiao Meng

**Affiliations:** ^1^Key Laboratory of Cardiovascular Proteomics of Shandong Province, Department of Geriatric Medicine, Qilu Hospital of Shandong University, Jinan, China; ^2^State Key Laboratory for Innovation and Transformation of Luobing Theory, Key Laboratory of Cardiovascular Remodeling and Function Research, Department of Cardiology, Chinese Ministry of Education, Chinese National Health Commission and Chinese Academy of Medical Sciences, Qilu Hospital of Shandong University, Jinan, China

**Keywords:** pulmonary arteriovenous malformations, stroke, cerebral abscess, hypoxemia, treatment

## Abstract

Pulmonary arteriovenous malformations (PAVMs) are direct pulmonary artery-to-vein connections without pulmonary capillaries that result in intrapulmonary right-to-left blood shunts. Although most patients with PAVMs may be entirely asymptomatic, PAVMs can induce a series of complications involving the neurological, cardiovascular, and respiratory systems that can lead to catastrophic and often fatal clinical sequelae. In this study we review the available literature and summarize the reported PAVM-related complications among patients with PAVMs. The reviewed studies included observational studies, case studies, prospective studies, and cohort studies, and we provide an overview of PAVM-related neurological and cardiopulmonary manifestations, including stroke, cerebral abscess, transient ischemic attack, cerebral hemorrhage, migraine, seizure, dizziness, cardiac failure, arrhythmia, myocardial infarction, cough, hypoxemia, dyspnea, respiratory failure, hemoptysis, and hemothorax. Identifying and treating PAVMs before the presentation of major complication is important because this can prevent the occurrence of complications and can result in better outcomes. PAVM patients should thus be better evaluated and managed by a multidisciplinary team because they may be in a treatable phase prior to their condition becoming life-threatening.

## 1 Introduction

Pulmonary arteriovenous malformations (PAVMs) are rare pulmonary vascular malformations that were first reported in an autopsy study by Churton et al. (1987) in Saboo et al. (1). PAVMs are estimated to affect approximately 1 in 2,600 individuals worldwide (2, 3). PAVMs are often detected in adults, although the disease may develop during childhood (4). Moreover, sex differences exist in the prevalence of PAVMs, occurring twice as often in females compared to males, but there is a male predominance in newborns (5).

**Figure 1:**
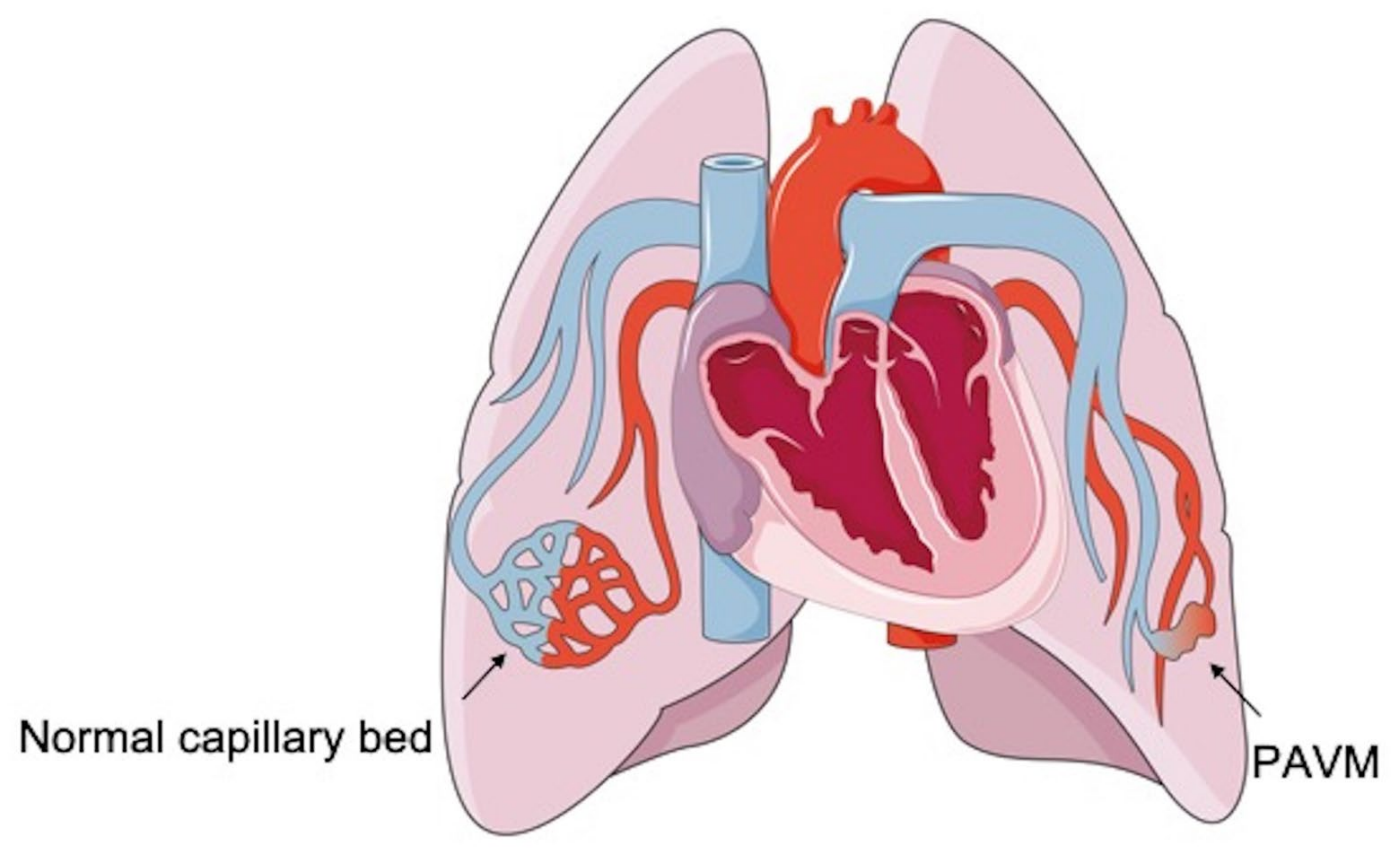
The structure of the normal capillary bed and the PAVM.

PAVMs are low-pressure and high-flow abnormal vascular structures that are characterized by a direct pulmonary artery-to-vein connection, thereby bypassing the normal pulmonary capillary bed (1, 6). These lesions can lead to an anatomic intrapulmonary right-to-left blood shunt because of the lack of a proper capillary network between the pulmonary arterial branch and the pulmonary venous tributary in the lungs, where blood bypasses the network that functions in gas exchange and filtration (Figure 1) (1, 7). Although PAVMs do not have malignant potential, they tend to increase in size over time because of pressure effects that gradually increase the intraluminal arterial blood flow (8–10). Moreover, women older than 60 years tend to have larger PAVMs than younger women (3).

## 2 The etiology and classification of PAVMs

Although the etiology of PAVMs is not well understood, most PAVMs (80–90%) are congenital in nature and occur as either an isolated abnormality or as part of hereditary hemorrhagic telangiectasia (HHT) (1). HHT, also named Rendu–Osler–Weber disease, is an autosomal-dominant familial disease with an estimated prevalence of 1 in 5,000, and it is most commonly caused by mutations in ENG, endoglin (HHT1), ACVLI/ALK1 (HHT2), or Smad4 (HTJP) (1). Epistaxis, gastrointestinal bleeding, and visible mucocutaneous telangiectasia are the major clinical manifestations in HHT patients (11–13). HHT can result in vascular malformations in many organs, including the lung, liver, and brain, particularly affecting the pulmonary vasculature and leading to PAVMs (7). It is estimated that about 70% of patients with PAVMs eventually manifest with HHT (5). HHT is considered to be the underlying cause of the PAVMs, which occur in 20–30% of the HHT population (14, 15). Compared to isolated forms, HHT-associated PAVMs are always multiple, bilateral, and have a slight preference for the lung bases (1). Moreover, HHT patients always have a rapid progression of PAVMs (16). International guidelines recommend that PAVMs should be screened for in patients with possible or confirmed HHT (15). Interestingly, there is a racial disparity in the presence of PAVMs among patients with HHT. Recently, a retrospective study showed that patients who identified as Asian have higher rates of PAVMs compared with patients who identified as white (17). Therefore, the association between race and PAVM incidence may be important for identifying risk factors in certain patient populations.

**Figure 2:**
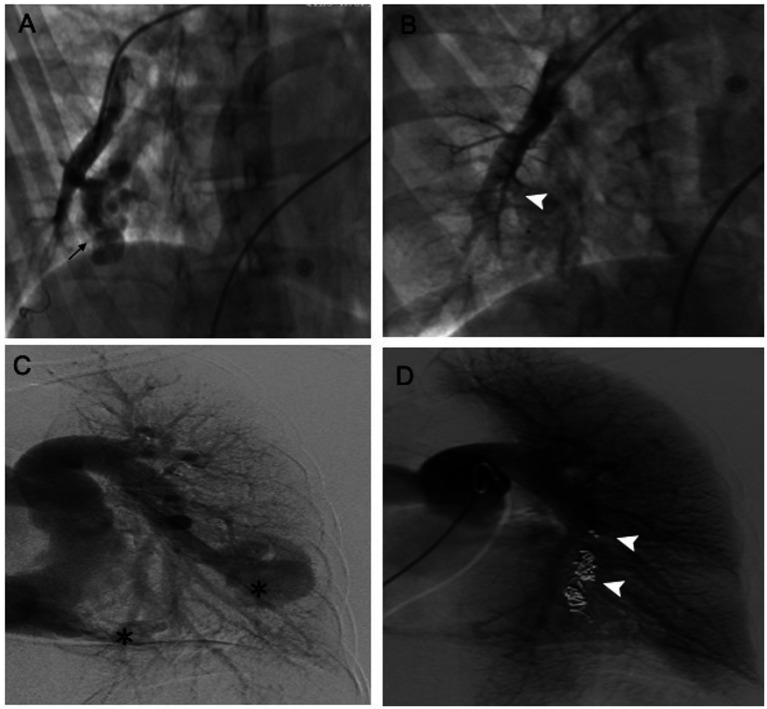
Cases of a simple and a complex PAVM before and after embolotherapy. **(A,B)** The simple PAVM was treated with Amplatzer Duct Occluder II (5 × 4 mm). **(C,D)** The complex PAVM was treated with Amplatzer vascular plug (10 × 7 mm) and Boston scientific interlock coils. (Black arrow, simple PAVM; asterisk, complex PAVMs; white arrowhead, embolotherapy material).

PAVMs are classified as unilateral or bilateral, single or multiple, and simple or complex in occurrence on the basis of their distribution and angioarchitecture (Figure 2). Most cases of PAVMs are multiple, and in one small-sample cross-sectional study including 75 patients with PAVMs, 49 patients (65.3%) had multiple PAVMs whereas 26 (34.7%) had single PAVMs (18). In another relatively large cohort of 170 patients, 73 patients (42.9%) had single PAVMs, 49 (28.8%) had multiple PAVMs, 36 (21.2%) had disseminated PAVMs, and 12 (7.1%) had diffuse PAVMs (19). Both solitary and multiple PAVMs are mostly located in the lower lobes of the lungs, with more presenting in the left lower lobe than in the right (6). In addition, according to the number of feeding pulmonary arteries, PAVMs can be divided into two types: the simple form consists of a lesion with a single feeding artery and single draining vessel, and these make up the majority of lesions (80–90%), while the complex form is characterized by two or more afferent and efferent vessels and is rare (2, 19, 20).

## 3 The diagnosis of PAVMs

**Figure 3:**
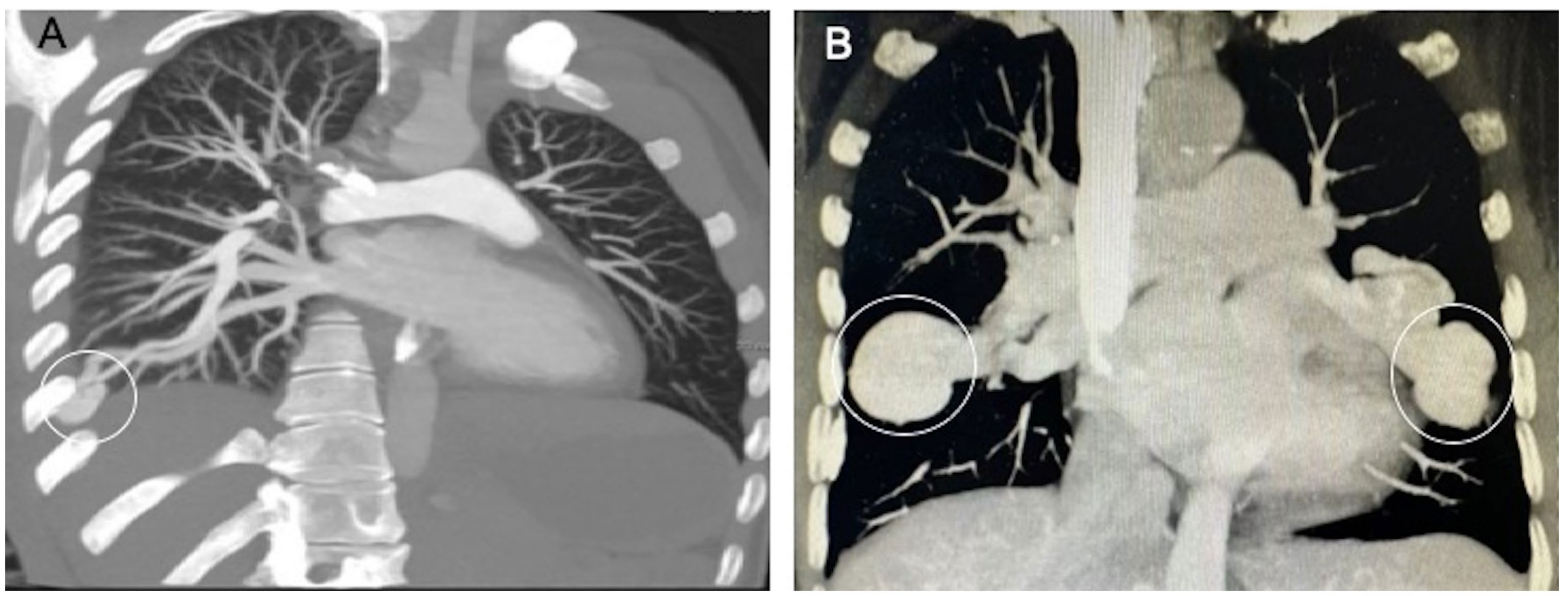
Typical CT appearance of a simple and a complex PAVM. **(A)** The simple PAVM. **(B)** The complex PAVMs. (White circle, aneurysmal sac).

PAVMs are not easily or routinely diagnosed because of their rarity and their unspecific clinical manifestations. Chest X-ray, computed tomography (CT) and magnetic resonance are the main tools for diagnosing PAVMs. Among these diagnostic tools, CT can provide a better anatomical definition of both pulmonary parenchyma and vascularization and is generally considered the “gold standard” for diagnosing PAVM (Figure 3) (21, 22). In one retrospective multicenter study, PAVMs were visible on CT scans in all patients, while only 54% of patients had visible PAVMs in chest X-ray images (23). Compared to digital subtraction pulmonary arteriography, CT has a greater sensitivity (83% vs. 70%) in the detection of PAVMs, although the specificity is less (78% vs. 100%) (24). Moreover, there are issues with the noninvasiveness of the procedure and the less radiation exposure when using CT. Transthoracic contrast echocardiography (TTCE) is a new diagnostic modality that has the ability to determine the grade of shunting and thus can predict the size of PAVMs and the subsequent feasibility for therapy. Among a cohort of 772 patients with possible or definite HHT, the positive predictive value of TTCE for the presence of PAVMs on chest CT was 13.4, 45.3, and 92.5% for pulmonary shunts of grade 1, 2, and 3, respectively (24). Grade 1 shunting chest CT can be avoided following TTCE because the PAVMs may be too small to be treated by embolization (25).

## 4 Comorbidities

From a clinical perspective, most patients with PAVMs may be entirely asymptomatic, especially with a single PAVM of less than 2 mm in diameter (14). However, some patients may present with a variety of clinical manifestations such as chest pain, cough, dyspnea, palpitation, and hemoptysis when the diameter of the PAVM is more than 2 mm or the diameter of the feeding artery is greater than 3 mm (26). The symptoms and signs of PAVMs mainly depend on the number, size, and shunt through the PAVM. Although some small PAVMs are silent, PAVMs may develop acute complications and lead to chronic disorders that result in adverse clinic outcome. A series of studies have shown that PAVM-associated complications are always multi-system and heterogeneous in their presentation, affecting the central nervous, cardiovascular, respiratory, and hematological systems. Importantly, PAVMs of any size are all associated with significant morbidity and mortality (27), and there is evidence to suggest that 50% of patients with PAVMs will develop disabling or fatal complications if left untreated (28). However, PAVMs are usually silent and are not noticed until further complications (1). Therefore, prompt diagnosis and correct management is needed when there is clinical suspicion of PAVMs (22). Preventing the progression of PAVMs and avoiding the risk of serious complications is a primary strategy for the treatment of PAVMs. In order to better understand this disease, we reviewed the available literature and summarized the reported PAVM-related complications.

### 4.1 Neurological disorders

Patients with PAVMs have a higher risk of developing embolic complications due to the right-to-left shunt, and PAVM-associated neurological symptoms are common. It has been reported that 2.4–58.7% of PAVM patients develop a neurological manifestation, including stroke, cerebral abscess, transient ischemic attack (TIA), migraine, and hemorrhage (Table 1) (18, 19, 23, 29–46), which are attributed to hypoxemia and paradoxical embolisation (6, 10, 47). Among them, ischemic strokes and brain abscesses affect greater numbers of individuals with PAVMs. In one retrospective cohort study including 219 patients diagnosed with PAVMs, 33.8% (*n* = 74) of the patients had a stroke or brain abscess (29). Importantly, many patients experienced ischemic stroke or brain abscess prior to the diagnosis of PAVMs (29). Considering that neurological complications represent the main causes of morbidity in patients with PAVMs, recognizing which PAVM patients are at risk of neurological complications is critical to facilitating appropriate management strategies for the treatment of PAVMs.

**Table 1:** Summary of the prevalence of neurological disorders in major PAVM series.

Study	*N* (female)	Manifestation	Prevalence
2005, Post et al. (32)	208 (136)	Stroke	16.8% (35)
2005, Post et al. (32)	208 (136)	TIA	8.7% (18)
2020, Albitar et al. (41)	77 (46)	Stroke/TIA	11.7% (9)
2000, Moussouttas et al. (18)	75 (53)	Stroke	45.3% (34)
2013, Angriman et al. (42)	88	Stroke/TIA	34.1% (30)
2018, Etievant et al. (19)	170 (92)	Stroke	16.5% (28)
2008, Shovlin et al. (29)	219 (140)	Stroke	13.7% (30)
2014, Shovlin et al. (30)	497 (302)	Stroke	12.3% (61)
2013, Velthuis et al. (39)	472	Stroke/TIA	7.2% (34)
2007, Cottin et al. (23)	126 (79)	Stroke/TIA	15.9% (20)
2000, Faughnan et al. (43)	16 (10)	Stroke/TIA	50% (8)
2001, Maher et al. (31)	71	Stroke/TIA	29.6% (21)
2022, Na et al. (44)	55 (49)	Stroke/TIA	20% (11)
2015, Kim et al. (33)	90 (73)	Stroke	15.6% (14)
2004, Mager et al. (40)	112 (68)	Stroke/TIA	14% (15)
2006, Pollak et al. (34)	154 (90)	Stroke	32.5% (50)
2006, Pollak et al. (34)	154 (90)	TIA	22.7% (35)
2010, Shin et al. (35)	23 (21)	Stroke	4.3% (1)
2008, Pierucci et al. (45)	36 (21)	Stroke/TIA	27.8% (10)
2021, Kroon et al. (46)	194 (108)	Stroke/TIA	13% (25)
2018, Fatania et al. (36)	30	Stroke/TIA	55.2% (17)
2018, Etievant et al. (19)	170 (92)	Cerebral abscess	8.8% (15)
2008, Shovlin et al. (29)	219 (140)	Cerebral abscess	12.8% (28)
2001, Maher et al. (31)	71	Cerebral abscess	7.0% (5)
2014, Kjeldsen et al. (37)	117	Cerebral abscess	7.8% (9)
2017, Boother et al. (38)	445	Cerebral abscess	8.3% (37)
2000, Moussouttas et al. (18)	75	Cerebral abscess	13.3% (10)
2005, Post et al. (32)	538	Cerebral abscess	3.5% (19)
2007, Cottin et al. (23)	126	Cerebral abscess	19% (24)
2005, Post et al. (32)	538	Migraine	16.4% (88)
2000, Moussouttas et al. (18)	75	Migraine	58.7% (44)
2001, Maher et al. (31)	71	Migraine	25.4% (18)
2000, Moussouttas et al. (18)	75	Seizure	14.7% (11)
2007, Cottin et al. (23)	126	Cerebral hemorrhage	2.4% (3)

#### 4.1.1 Stroke

The brain is the most susceptible organ to circulatory abnormalities, and stroke can have serious and disastrous consequences. Patients with PAVMs are at increased risk for developing ischemic stroke, and stroke is by far the most common neurological complication of PAVMs because of paradoxical embolism (1, 48). These paradoxical microemboli unfiltered by the pulmonary capillary network enter the cerebral circulation through the right-to-left shunt, and can result the occlusion of cerebral arteries, which has been widely accepted as a potential cause of acute ischemic stroke (48, 49). PAVMs have been confirmed to constitute a minority of ischemic stroke admissions, and data analyses have shown that 0.02% patients admitted to the hospital with ischemic stroke have PAVMs (50).

Based on a series of epidemiologic studies, approximately 4.3–45.3% of PAVM population experience proven ischemic stroke (18, 19, 29–35) (Table 1). Interestingly, PAVM-related ischemic stroke presents in a younger demographic. In a large retrospective study enrolling 4,271,910 patients with acute ischemic stroke, patients with both stroke and PAVMs were younger than those patients without PAVMs (57.5 years vs. 72.5 years), suggesting that PAVM-related stroke may be independent of other strokes in terms of clinical characteristics (50). Moreover, stroke patients with PAVMs had lower age-adjusted crude inpatient morbidity (39.6% vs. 46.9%) and in-hospital case fatality rates (1.8% vs. 5.1%) (50). Also, in another cohort study enrolling 497 patients with CT-proven PAVMs due to HHT, 61 patients (12.3%) had acute, non-iatrogenic ischemic clinic stroke at a younger age (median age of 52 years) (30). In addition, the stroke distribution type is different among these patients. The majority of patients (70.5%) had partial anterior circulation syndromes, while fewer had partial posterior circulation syndromes (27.9%) or lacunar circulation syndrome (1.6%) (30). However, there was no difference in ages between the stroke distribution types.

Patients with multiple PAVMs always have a higher incidence of cerebral infarct compared to patients with single PAVMs (18). In one retrospective cross-sectional study, Moussouttas et al. (18) found that the incidence of cerebral infarction increased from 32% in patients with a single PAVM to 60% in those with multiple PAVMs among a cohort sample including 26 single PAVMs and 49 multiple PAVMs. These significant differences in infarction prevalence between the two groups suggested an increased predisposition to neurologic complications in patients with greater numbers of malformations.

Silent cerebral infarction (SBI) is characterized by the incidental finding of cerebral infarction on imaging without any clinically apparent neurological deficit (51). However, SBI is not a completely silent and benign event, and mounting evidence suggests that multiple SBIs independently increase the risk of recurrent ischemic stroke (52). These lesions are frequently associated with neurological deficits and early mortality (51). Among PAVM patients, the burden of SBI is more than that of clinical stroke and should not be ignored. In a recent small study including 29 PAVM patients with no previous history of clinical stroke, 16 patients (55.2%) had between one and five SBIs assessed by magnetic resonance38. Moreover, the most frequently affected sites were the cerebellum (40%) and thalamus (14.3%) (36). Further studies were performed by Brinjikji et al. (53), who found that the prevalence of SBIs increased with age, affecting 0, 10, 28.6, and 27.6% of patients with PAVMs who were <30, 30–39, 60–69, and ≥70 years old, respectively, while they affected 0, 0, 10.5, and 10.5%, respectively, in patients without PAVMs. Thus, the patients with PAVMs experience a higher prevalence of SBIs than the general population, particular in patients over the age of 60 years old. Multivariate analysis indicated that only PAVMs and increasing age are independently associated with SBI (53). Evidences showed paradoxical embolism is involved the pathogenesis of SBI (51, 54). In the ICONS study, the prevalence of right-to-left shunt among those individuals with SBI located outside the perforating artery territory was similar to that of cryptogenic embolic stroke (51% vs. 62%) (54).

Based on prior studies, PAVMs appear to be a novel risk for ischemic stroke. Therefore, recognizing the higher risk of stroke in order to facilitate appropriate management strategies among PAVM patients is particularly important. In the PAVM population, the risk of ischemic stroke may be attributable not to conventional vascular risk factors, but to the severity of PAVMs. Previous studies showed that there was no association between the conventional neurovascular risk factor and ischemic stroke in patients with PAVMs (2). Attention then focused on the diameter of the feeding artery and the degree of right-to-left shunt, which is considered to be associate with an increased risk of neurological complications in patients with PAVMs. Etievant et al. (19) analyzed the data from a total of 170 patients with PAVMs and found an association between the mean diameter of the largest feeding artery of the PAVMs and ischemic stroke frequency, suggesting that a larger feeding artery is more strongly related to the risk of ischemic strokes. However, there have been some conflicting reports, and there is evidence that stroke risks cannot be predicted by respiratory symptoms or PAVM severity in PAVM patients. In the study by Shovlin et al. (29), they did not find an association between the primary determinants of stroke risks and PAVM severity. In addition, Moussouttas et al. (18) showed that the prevalence of cerebral infarction did not appear to be related to larger feeding artery diameters when the diameters exceeded 3 mm in patients with single PAVMs, and no increased risk for cerebral infarction was seen when the feeding artery was less than 3 mm. Based on these findings, other factors might be involved in the pathogenesis of severe neurological manifestations in patients with PAVMs. Prior studies have reported that patients with PAVMs combined with iron deficiency may have an increased risk of ischemic stroke. In one retrospective cohort study of 497 patients with CT-proven PAVMs, ischemic strokes were more common in those patients with low serum iron. Among PAVM patients, the risk of stroke was approximately double for a serum iron concentration of 6 μmol/L compared to a serum iron level in the mid-normal range (7–27 μmol/L), suggesting that stroke risk may be inversely associated with low serum iron rather than with venous thromboemboli, conventional neurovascular risk factors, or the severity of the right-to-left shunt (2). The pathogenesis may be associated with enhanced platelet aggregation responses induced by iron deficiency. Interestingly, ischemic stroke appeared more common in patients who had experienced at least one myocardial infarction. Even after adjusting for all other measured variables, this association persisted, suggesting the involvement of paradoxical embolic aetiology (2).

The use of antiplatelet agents for the prevention of ischemic stroke caused by PAVMs has been recommended in guidelines (55). Although there might be a higher risk of nosebleeds, patients with HHT/PAVMs tolerate antiplatelet or anticoagulant therapy better than expected (56). In addition, the benefit of therapeutic PAVM occlusion in preventing the recurrence of ischemic events in PAVM patients with stroke has been established (16).

The presence of patent foramen ovale (PFO) also provides a channel to permit the right to left shunting of embolic material to the brain, and PAVMs are often misdiagnosed as PFO. In clinical practice, PAVMs and PFO may be coexist in some patients (49, 57, 58). The existence of an isolated PAVM in patients with cryptogenic stroke should not be overlooked, even if a PFO is present. PAVMs can arise as residual defects after PFO closure, and it has been reported that some patients have recurrent cerebrovascular events after the closure of a PFO (49, 57).

Considering the prevalence of PAVMs in the population, it can be estimated that PAVMs should be responsible for a significant portion of stroke patients. It has been reported that approximately 1 in 1,000 acute ischemic stroke hospitalizations can be attributed to a PAVM, while only 1 in 5,000 was captured (59). Therefore, unexplained cryptogenic stroke should raise the suspicion of the presence of PAVMs, and thus a better understanding of risk factors for PAVM patients who may develop stroke is important.

#### 4.1.2 Cerebral abscess

Cerebral abscesses always leads to unfavorable prognosis, including death, and among those who survive 80% of patients are unable to return to their former occupation because of persistent neurological deficits (29). Accumulating evidence suggests that patients with PAVMs have a considerable risk of suffering from cerebral abscesses. A series of epidemiological studies showed that about 3.5–19% of PAVM patients are complicated with cerebral abscess, often located at the basal ganglia (18, 19, 23, 29, 31, 32, 37, 38, 60). In general, most PAVM patients experience a cerebral abscess within their first 50 years of life (38). However, the majority of PAVM-induced cerebral abscesses may be the initial clinical manifestation, and these commonly occur in asymptomatic subjects who have not yet received a diagnosis of PAVMs (29, 38). There is always a delay between the abscess event and later PAVM diagnosis, so patients with unexplained cerebral abscess should be evaluated for PAVMs (37).

PAVM patients with cerebral abscesses are more likely to have multiple PAVMs. A study showed that the prevalence of brain abscesses in patients with multiple PAVMs was twice that compared to patients with a single PAVM (18). However, considering the small sample of only 75 individuals in that study, more promising results might be obtained when the sample size is enlarged. In PAVM patients, the cerebral abscesses may be isolated or multiple (15). Of these, most cases are multiple or recurrent, which could lead to substantial morbidity and healthcare burdens in PAVM patients (31). In one retrospective cohort study including 445 patients diagnosed with PAVMs, 37 patients (8.3%) experienced a cerebral abscess and all required neurosurgery, longer antibiotic therapy, and prolonged inpatient hospital stays. Moreover, more than half of the cases (19 of 37 cases, 51.4%) suffered residual life-changing neurological deficits, most commonly including memory loss or other cognition impairment, hemiparesis, and visual defects (38).

The pulmonary capillary bed normally provides a first-pass filtration system that can remove small thrombi and bacteria before they enter the circulation. Because of the lack of proper filtration by the pulmonary capillary bed in PAVMs, the capacity to eliminate bacteria or infected material is damaged. Thus the bacteria or infected material are able to pass through the PAVM and into the cerebral circulation, which can result in an abscess (7, 39). Recently, the association between PAVM-induced cerebral abscess and microbial species has been a focus of research. Boother et al. (38) found that bacterial species cultured from PAVM patients with cerebral abscesses were microaerophilic and anaerobic bacteria predominantly of odontogenic origin.

Recently, a linear association between low oxygen saturation (indicating greater right-to-left shunt) and the risk of cerebral abscesses has been established among PAVM patients (38). For each 1% rise in oxygen saturation, the risk of cerebral abscess is reduced by 10.47% (38). It is expected that the risks of neurotic compilations (including stroke and cerebral abscess) can be evaluated based on the severity of right-to-left shunting in PAVM patients. Recently, Velthuis et al. (39) showed that the presence of cerebral manifestations (including ischemic stroke, TIA, or cerebral abscess) differed significantly according to pulmonary shunt grade (grade 0, 1, 2, and 3 pulmonary shunt corresponding to 1.4, 0.4, 6.5, and 20.9%, respectively) in patients screened for HHT, suggesting a striking association between pulmonary right-to-left shunt grade and the prevalence of cerebral complications. Moreover, pulmonary shunt grade 2 and 3 are both independent predictors for the prevalence of a cerebral ischemic event or abscess. However, some conflicting results have been also reported. In one study by Etievant et al. (19), an increased risk of cerebral abscess was found in those patients with multiple, diffuse, or disseminated PAVMs, but no increased risk with increased severity of the right-to-left shunt, indicating an association between the number of PAVMs and the prevalence of brain abscesses. Another contradicting study was that of Velthuis et al. (39), where the risk of cerebral abscess failed to be predicted by the size, severity, or symptoms of PAVMs in patients. They argued that there was no significant relationship between cerebral abscess and PAVM severity markers (29). Moreover, another study found no relationship between cerebral abscess risk and other PAVMs-related neurological complications (such as ischemic stroke and migraine headaches), variables associated with ischemic stroke risk, or the diameter of the largest PAVM-feeding artery (38). Based on these conflicting results, whether shunt size and PVAM severity should be taken into consideration when evaluating the potential risk of neurological complications requires further study.

In addition, males with PAVMs present with higher cerebral abscess rates (29, 38). In one retrospective cohort study, the majority (60.7%) of PAVM patients with brain abscesses were male (29). In another retrospective study analyzing the data from a total of 445 patients with confirmed PAVMs, multivariate logistic regression showed that the prevalence of cerebral abscess was associated with male sex and was estimated to be 2.63-fold higher than in females and was also associated with low oxygen saturation and venous thromboemboli. Unexpectedly, the risk of cerebral abscess was greater in patients with a higher transferrin iron saturation index and in those receiving intravenous iron supplementation (38).

It is important to screen for patients with PAVMs who are at risk for cerebral abscess even though the majority of individuals are asymptomatic. For patients with confirmed cerebral abscesses, prompt and full antibiotic treatment is needed. In addition, antibiotic prophylaxis for procedures with a risk of bacteremia is also be recommended due to the potential risk of cerebral abscess (6).

#### 4.1.3 TIA

A TIA is an important predictor of stroke, and TIAs and stroke share similar pathophysiologic mechanisms to some extent. Of note, the 90-day risk of stroke may be up to 17% after a TIA (55). Moreover, the greatest risk is in the first week. In most instances TIA and stroke have identical preventive strategies, but the prognosis may vary depending on severity and cause (55).

PAVM patients can experience TIAs. In one retrospective multicenter study enrolling 126 patients with PAVMs, 35% patients had ischemic central nervous system events and TIA attack was reported in 6.3% of patients (23). Also, in a total of 208 PAVM patients, a significantly higher prevalence of TIA was found compared to those without PAVM (8.7% vs. 1.2%) (32). Percutaneous embolization can effectively low the risk of TIA in PAVM patients. In a case study, a 65-year-old woman with a history of multiple TIAs was diagnosed with PAVMs and received catheter closure. Over 2 years of follow-up, the patient did not have any further episodes of TIA or stroke and her neurological symptoms were completely resolved (61). Although some PAVM patients successfully received embolotherapy, they might experience TIA during follow-up because of recanalization of PAVMs (40). Patients should remain under regular review after embolotherapy.

#### 4.1.4 Cerebral hemorrhage

Among PAVM patients, the risk of cerebral hemorrhage is low compared with other cerebral events (stroke, cerebral abscess, and TIA). Prior studies have reported that cerebral hemorrhage is only present in 2.4% of patients and accounts for only 5.7% of central nervous system events in PAVM patients (23). Of note, these malformations appeared more often in PAVM patients combined with HHT.

#### 4.1.5 Migraine

As a common neurovascular brain disorder, migraine seriously affects individuals’ quality of life and results in significant economic burden. However, the symptoms and clinical features of migraine are complex and variable, and the pathogenesis remains poorly understood. Therefore, many different treatment strategies have been implemented. The relationship between cardiopulmonary pathology and migraine has been illustrated by a series of studies, and an association between migraine and right-to-left shunt has been established (62). Abnormal shunting can lead to the passing of paradoxical microemboli through the open physiological channels in PAMVs and lead to production-inactivation imbalances of some neurotransmitters that are released into circulation instead of being trapped in the pulmonary capillaries (26, 63). The microemboli and/or chemical substances in venous blood may initiate cortically spreading depression and can trigger an attack of migraine when they arrive at the brain in sufficient concentrations through the abnormal shunt, which establish a plausible link between PAVMs and migraine (62, 64, 65). For example, 5-hydroxytryptamine (5-HT), which is normally eliminated on passage through the lungs, has been reported to be involved the pathogenesis of migraine (66). Drugs that influence 5-HT receptors have been reported to be used in treating migraine (66).

A higher prevalence of migraine headache symptoms in PAVM patients has been reported in prior cohort studies, with an incidence of 4–38% (6). In one study by Maher et al. (31), among 71 patients with PAVMs, 18 (25.4%) had a known history of migraine headaches. Given that approximately 15% of the general population globally are affected by migraine (67), this finding might represent an increase over the normal incidence of migraine headache. Kakehi et al. (65) reported a 40-year-old woman with a history of migraine for 30 years who was diagnosed with PAVMs. A case of PAVMs in a woman aged 41 years who suffered from migraine with optic aura once or twice every month for over 20 years was also presented in the study by Kakeshita et al. (68). After transcatheter embolization, the two patients had no episodes of migraine. Although the migraine was resolved in the two patients after successful closure of the PAVMs, the effectiveness of PAVM closure in treating migraine has not been confirmed. Anti-platelet therapy also achieved significant therapeutic efficacy in some cases. For example, Onorato et al. (69) described a 38-year-old woman suffering from episodes of migraine with aura caused by an isolated PAVM, and after thienopyridine therapy, not embolization, the migraine symptoms were almost completely resolved. Therefore, further population-based studies are needed to develop appropriate strategies for treating migraine in patients with PAVMs.

Numerous clinic-based studies have shown that patients with migraine among all population groups are at higher risk for stroke (67). In a large Danish study enrolling 51,032 patients with migraine over 19 years of follow-up, the risk of ischemic and hemorrhagic stroke was significantly increased compared with the general population, suggesting that migraine is an important risk factor for stroke (70). Recently, Zhang et al. (63) found that migraine patients with grade 2 or 3 right-to-left shunting may be more likely to develop stroke. However, that study focused on PFO-related right-to-left shunt, not PAVM-related right-to-left shunt. Considering the difference between the two diseases, whether those PAVM patients with migraine who have higher grades of right-to-left shunting are at greater risk of suffering a stroke requires more study.

#### 4.1.6 Seizure

Seizure is frequently seen in patients with PAVMs. Moussouttas et al. (18) analyzed the data from 75 consecutive patients admitted to Yale-New Haven Hospital between 1988 and 1992 for treatment for PAVMs, and they found that 14.7% of patients developed seizure. The incidence of seizures in patients with multiple PAVMs was at least twice that in patients with a single PAVM. Moreover, seizures in patients with multiple PAVMs were associated with the occurrence of cortical infarctions and brain abscesses.

#### 4.1.7 Dizziness

Simple PAVM-related dizziness is rare and is only mentioned in sporadic case reports. In one study by Cao (71), a 73-year-old woman experienced recurrent dizziness for more than 10 months without hypoxemia, hemiplegia, or altered consciousness, and a PAVM was found in her left upper lobe. After percutaneous PAVM embolization, the patient’s dizziness was relieved without recurrence. However, the patient had a history of hypertension and atherosclerosis and received anti-platelet and blood pressure-lowering therapy, so medical treatment cannot be entirely excluded in eliminating the dizziness.

Brain manifestations are frequent and dangerous in PAVM patients. Considering that neurological PAVM-associated risks are common, PAVM patients should be screened and aggressively managed, even for asymptomatic PAVMs. Surprisingly, there is no significant difference in the risk of central nervous system complications in patients with single and with multiple PAVMs, suggesting that similar attention should be paid to patients with single and multiple PAVMs (23). In PAVM patients, the risk of ischemic stroke and cerebral abscess can be reduced after embolization. Unfortunately, clinical practice guidelines addressing the acute management and secondary prevention of neurological syndromes (ischemic stroke, cerebral abscess, and TIA) in patients with PAVMs remain rare (59). Whether these PAVMs should be treated once they are discovered requires further study.

### 4.2 Cardiovascular disorders

#### 4.2.1 Cardiac failure

In general, PAVMs do not influence cardiac hemodynamics. However, patients may be symptomatic when the right-to-left shunt is greater than 20% of the systemic cardiac output (72). Increased cardiac output can result in heart failure (73, 74). Liao et al. (73) described an unusual case presenting with refractory heart failure with a moderately dilated left ventricle and significantly decreased left ventricle ejection fraction caused by PAVMs.

Pulmonary load is a central determinant of right ventricular (RV) systolic function. The RV dysfunction can result in the dilatation of right ventricle and subsequent heart failure (75). The occurrence of pulmonary arterial hypertension (PAH) is associated with RV dysfunction. In theory, the development of PAVMs may decrease the pulmonary arterial pressure and unload the right ventricle, and PAVM embolism may increase the pulmonary arterial pressure and RV afterload, thus predisposing the patients to right heart failure in the presence of PAH (76). However, heterogeneous results have been published. Shovlin et al. (77) studied 143 cases and showed that there was no significant increase in pulmonary artery pressure or right heart failure as a result of PAVM embolisation. It should be noted, however, that patients with severe PAH were excluded from the study. Thus, more studies are needed to predict the outcomes of patients with cardiac involvement.

#### 4.2.2 Arrhythmia

Some patients with PAVMs present with arrhythmia. As documented by the study by Santhirapala et al. (78), 29% (74 of 257) of PAVM patients had postural orthostatic tachycardia, and this was more pronounced in patients with orthodeoxia than in patients without orthodeoxia. The occurrence of orthostatic tachycardia may be part of acute compensatory mechanisms that maintain tissue oxygen delivery when the arterial oxygen content is falling (78).

#### 4.2.3 Myocardial infarction

PAVMs can manifest as cardiac ischemic events due to paradoxical embolism. In a single-center study enrolling 98 patients with PAVMs, 6 patients experienced typical angina pectoris-like chest pain or had a myocardial infarction before PAVM embolotherapy (79). The occurrence of cardiac ischemia may be caused by a paradoxical embolus passing through the PAVMs to a coronary artery (79).

### 4.3 Pulmonary disorders

#### 4.3.1 Hypoxemia and dyspnea

In PAVMs, blood flows directly from the pulmonary artery to the pulmonary vein because of the right-to-left shunt, thus bypassing the capillary-alveolar barrier with no effective gas exchange. Therefore, PAVMs impair normal gas exchange and can lead to hypoxemia and dyspnea as well as enhanced ventilatory demands (2). The impairment of gas exchange mainly depends on the size of the PAVMs (27). A smaller physiologic burden in intrapulmonary right–left shunt does not affect blood oxygenation (80), Therefore, PAVM patients rarely present with dyspnea or respiratory syndromes and are usually asymptomatically hypoxemic (81). The degree of right-to-left shunt determines the severity of hypoxemia, with severe hypoxemia occurring when the shunt is more than 20% of the cardiac output (6). Thus, serious hypoxemia may appear in patients with more diffuse and severe types of PAVMs (6). In addition, orthodeoxia is often noted due to PAVMs that are commonly located in the lower lobes, middle lobes, and the lingula (1, 21, 82). Santhirapala et al. (78) enrolled 258 patients with PAVMs and assessed their postural changes using validated pulse oximetry methods. They found that 29% of the patients had orthodeoxia with an oxygen saturation drop of at least 2% on standing. Acute drops in oxygen saturation will induce a decrease in the arterial oxygen content per unit blood volume. Fortunately, PAVM-associated platypnea is rarely experienced, suggesting that acute drops in arterial oxygen content can be successfully compensated for in the PAVM population (78). Moreover, pulmonary vascular resistance at rest is low in PAVM patients, and hypoxemic PAVM patients are not considered to be at risk of hypoxic pulmonary hypertension (83).

Although long-term oxygen therapy can be used to improve hypoxemia, whether it is beneficial in PAVMs patients remains controversial. Moreover, there is no evidence that supplemental oxygen application can decrease the risk of PAVMs-related complications, although oxygen therapy may of course be indicated when PAVM patients have comorbidities such as neurological and cardiovascular disorders (84). After embolization, the patients will have a secondary erythrocytic response and restored oxygen saturation (81, 83). Moreover, this improvement will persist for a long period. However, the majority of patients have no change in exercise at their post-embolization follow-up (83). Hypoxemic patients still maintain normal oxygen delivery/consumption during peak exercise in the PAVMs patient population, utilizing both secondary erythrocytosis and the maintenance of the oxygen pulse (81).

#### 4.3.2 Hemoptysis and hemothorax

PAVMs occasionally manifest as a clinical emergency with hemoptysis or hemothorax caused by PAVM-associated hemorrhage (85). Moreover, these patients are always complicated with HHT. In PAVMs, the angioarchitecture between the pulmonary artery and the pulmonary vein is fragile and may rupture and bleed as the PAVM size increases (6). The incidence of PAVM rupture ranges from 2 to 8% (86). The rupture of PAVMs can induce hemoptysis or hemothorax because PAVMs are usually subpleural (1, 82). Elmali et al. (87) described a 51-year-old woman with sudden-onset chest pain who was diagnosed with PAVMs by thoracic CT angiography, and the patient underwent thoracentesis and hemorrhagic fluid was found. Although significant PAVM-associated pulmonary hemorrhages are a life-threatening complication, their incidence is rare. One prior multicenter study showed that 12% (15 of 126) of patients had hemoptysis, but only one patient was severe, and only 4 patients (3%) presented with hemothorax (23). Ference et al. (88) showed that only 11 patients (8%) developed a history of either massive hemoptysis or hemothorax in another retrospective study of 143 patients with PAVMs and HHT, and only one patient died. It is agreed that pulmonary hemorrhage caused by the rupture of the PAVMs should be treated aggressively with transcatheter embolotherapy or surgical intervention (88). However, which PAVMs are most likely to rupture remains difficult to predict.

The presence of PAH predisposes for the enlargement of PAVMs and subsequent rupture, but rupture generally only occurs in patients who have thin-walled PAVMs or in pregnant patients (76). Recent case reports highlighted the risk. A PAVM with a 5-mm diameter feeding artery in the right lower lobe was reported in a 29-year-old patient combined with HHT and PAH. Although hemodynamics were improved after dual endothelin receptor antagonist and inhaled iloprost, the patient died suddenly of a rupture of the PAVM into the pleural cavity (89). A good outcome was reported in a 28-year-old male presenting with an 8-year history of recurrent hemoptysis and a year of chest pain, which was confirmed as PAH and diffuse PAVMs. After treatment with sildenafil for 1 year, the symptoms of hemoptysis disappeared in this patient (90).

Although PAVM-associated hemorrhage is rare, it is the most common contributor to the 1% rate of maternal death in pregnancy (2, 91). The increased risk for rupture and hemorrhage in the pregnant population with PAVMs may be associated with increased cardiac output and decreased vascular wall stability (1). Pregnancy has been regarded as a hazardous period for women with PAVMs (79). Prior recognition of HHT or PAVM diagnosis and provision of additional care can contribute to improving survival outcome in women experiencing a life-threatening event (91).

#### 4.3.3 Cough

PAVMs can be responsible for the occurrence of chronic cough. However, this is only published in the case report-biased literature and lacks systematic reporting. In 2015, Jutant et al. (92) reported a 51-year-old female who suffered from severe chronic cough caused by PAVMs. After surgical intervention, the cough completely disappeared. Unfortunately, the exact mechanism through which a PAVM can cause coughing remains elusive. It is speculated that the irritation of the pleura due to the sub-pleural location of the arteriovenous malformations may be involved (92).

#### 4.3.4 Respiratory failure

PAVM patients rarely present with acute respiratory failure, and such presentations are limited to case reports. Recently, Nusca et al. (93) reported a 54-year-old male who was admitted for acute respiratory failure with reduced oxygen saturation (90%) and mild distal-limb cyanosis attributed to the presence of a voluminous PAVM. After successfully percutaneous embolization, the arterial saturation increased to 99%.

### 4.4 PAVM-related splanchnic vessels embolization

PAVMs are accompanied by paradoxical embolism in many organs due to the right-to-left shunt, thereby likely resulting in splanchnic vessel embolization. Prior studies reported that PAVMs can induce the occurrence of renal infarction, superior mesenteric arterial infarction, splenic infarction, and abscesses (94, 95). However, it is very rare for PAVMs to be complicated by splanchic vessel embolization, and most are sporadic cases (96).

## 5 Treatment

Not all PAVMs require treatment. However, PAMV patients with neurologic complications, hypoxemia, or having a feeding artery ≥3 mm in diameter are recommended for active treatment. Percutaneous transcatheter embolotherapy and surgery are the main choices of treatment strategy, and percutaneous embolotherapy of the feeding artery of PAVMs is currently the first-line therapy due to its effectiveness, high level of safety, reduced invasiveness, and shorter hospital stay and because it is associated with reduced loss of lung tissue and low risk of complication. Embolization is amenable in the majority of cases and has effectively replaced surgery. Prior studies have shown the long-term efficacy of embolization with detachable balloons, metallic coils, and Amplatzer vascular plugs in the treatment of PAVMs (61, 97). After successful closure of PAVMs, the patients usually have a good prognosis. However, although most cases are successfully treated, intervention strategies have the risk of recanalization, reperfusion, and downstream migration of the device with paradoxical embolism (92, 98, 99). Recanalization accounts for more than 90% of persistent PAVMs after embolotherapy and is also the most common mechanism of PAVM reperfusion (85, 100). Jutant et al. (92) reported a 51-year-old female with PAVMs who underwent embolization; however, reperfusion was present in the previously embolized fistula and surgical excision of the malformation was ultimately undertaken. Recently, Takao et al. (98) reported two cases that presented with cerebral infarction due to PAVM recanalization 13 and 30 years after the first coil embolization. Therefore, periodic follow-up is needed after catheter embolization. One retrospective study indicated that reperfusion may occur due to increased feeding artery diameter, an insufficient number of coils, the use of oversized coils, and proximal coil placement within the feeding artery more than 1 cm from the sac (85).

Besides endovascular embolization of the feeding artery, transcatheter venous sac embolization (VSE) of PAVMs using interlocking detachable coils is an alternative method to the arterial route (101, 102), and VSE may be beneficial in PAVMs with large out-flow vessels or short feeding arteries. Moreover, VSE may prevent the risk of systemic migration of embolic materials in PAVMs (101). In a small retrospective study by Hayashi et al. (102), 37 patients with PAVMs were enrolled to assess the efficacy of VSE (15 patients) and transcatheter feeding artery embolization (FAE, 22 patients) with coils for the treatment of PAVMs. Reperfusion occurred in 50% (11 of 22) of the patients in the FAE group and in no patients in the VSE group. Therefore, VSE is considered safe and to have long-term efficacy.

Despite surgical interventions requiring longer hospital stays and carrying a higher risk of complications, there are suggestions in the literature that minimally invasive anatomic lung resection by surgical treatment can be considered in select cases, especially for patients with diffuse lobar, segmental, solitary large PAVMs and for patients with complicated PAVMs or in cases of life-threatening bleeding after PAVM rupture (103, 104). Nagano et al. (105) performed a retrospective study among Japanese patients with PAVMs, including 211 cases who underwent surgery and 785 cases who underwent embolotherapy. Although a higher incidence of composite complications and prolonged postoperative hospital stay was noted in the patients who underwent surgery, surgery had a markedly lower rate of reintervention for PAVMs, suggesting that surgery had a higher curability compared to the patients who underwent embolotherapy. Next, Irie et al. (106) documented the safety and efficacy of video-assisted thoracic surgery in treating idiopathic peripherally located simple-type PAVM. In this study, 23 patients were treated successfully with surgical resection and no serious complications were observed. Recently, Li et al. (107) presented the case of a 44-year-old man with PAVM who had undergone transcatheter embolization three times but still relapsed. Moreover, he developed new symptoms of TIA after the third intervention. Fortunately, the patient received thoracoscopic surgery and no recurrence was observed. Despite transcatheter embolization being less invasive and being easier to repeat, repetitive interventional procedures increase the risk of potential complications, the duration of radiation exposure, and the dose of nephrotoxic contrast. In another study, a 55-year-old patient with a large PAVM complicated by hemothorax received transcatheter embolization. However, a subsequent emergency open thoracotomy was conducted due to active bleeding (108). A larger diameter of the feeding artery may be responsible for the failure of embolization, and therefore transcatheter embolotherapy cannot guarantee success for all patients, especially in complicated cases. Surgery may thus be a reasonable treatment option for PAVM patients who are difficult to treat with embolotherapy or in refractory cases who have repeatedly experienced failed interventional procedures (109).

Advances in catheter techniques have increased the proportion of patients who are eligible for embolotherapy. However, transcatheter embolization cannot completely replace surgical resection in all patients with PAVMs. Surgery as a choice of treatment strategy should therefore be considered in specific cases to achieve complete recovery. Altogether, current work in the field suggests that an individualized approach is necessary for the treatment of PAVMs.

## 6 Conclusion

PAVM patients are typically asymptomatic; however, they are at risk for a range of complications affecting the neurological, cardiovascular, respiratory, and hematological systems, which can sometimes lead to catastrophic and fatal outcomes. Therefore, screening for PAVMs in asymptomatic patients and implementing appropriate management strategies are crucial to prevent complications and improve outcomes, particularly since these patients may be in a treatable phase before the condition becomes life-threatening. Unfortunately, PAVMs are often not suspected until complications have already arisen. Consequently, identifying and treating PAVMs before major complications develop remains a significant challenge in clinical practice.

The management of PAVMs relies on a deeper understanding of the disease, which could facilitate the development of personalized therapeutic approaches. Our study reviews the etiology, classification, diagnosis, comorbidities, and treatment of PAVMs, with the goal of advancing current knowledge of the condition. An effective management strategy may reduce the incidence of complications in patients with PAVMs. Therefore, a coordinated multidisciplinary team, including respiratory specialists, thoracic surgeons, neurologists, cardiologists, hematologists, radiologists, epidemiologists, and geneticists, is often essential to prevent complications and optimize treatment for patients with PAVMs.
